# The relationship between depression and lipid accumulation product: a population-based study

**DOI:** 10.3389/fpsyt.2024.1395766

**Published:** 2024-07-08

**Authors:** Xianlin Zhu, Peng Wang, Ya Yue, Tiancheng Wu, Jiali Cui, Yanping Shu, Ling Ma

**Affiliations:** ^1^ Department of Clinical Psychology, The Third Affiliated Hospital of Soochow University, Changzhou, China; ^2^ Department of Neurology, Anqing Municipal Hospital, Anqing, China; ^3^ Department of Psychiatry of Women and Children, The Second People’s Hospital of Guizhou Province, Guiyang, China; ^4^ Department of Rehabilitation Medicine, Kangda College of Nanjing Medical University, Nanjing, China; ^5^ Ninth clinical Department, Mental Health Center, the First Hospital of Hebei Medical University, Shijiazhuang, China

**Keywords:** depression, NHANES, LAP, obesity, survey

## Abstract

**Background:**

Lipid Accumulation Product (LAP) is a new type of obesity index. The relationship between LAP and depression is unclear, and this cross-sectional study was conducted to explore the relationship between LAP and depression using the National Health and Nutrition Examination Survey (NHANES) database from 2005–2018.

**Methods:**

In our study, logistic regression analysis was used to calculate the odds ratio between depression and LAP, and subgroup analysis and sensitivity analysis were also performed to verify the robustness of the results.

**Results:**

The analysis included 13,240 participants aged 20 years or older. After adjusting for multiple variables, LAP was positively associated with depression, OR 1. 50 (95% CI, 1. 05–2. 12). In subgroup analysis, LAP was significantly positively, associated with depression among male (2. 52, OR; 95% CI, 1. 39,4. 57), non-Hispanic Black (2. 55, OR; 95% CI, 1. 49,4. 36), those without diabetes (1. 67, OR; 95% CI, (1. 06,2. 61) or in the overweight (2. 09, OR; 95% CI, (1. 23,3. 54) subgroups. After inverse probability of treatment weighting (IPTW), the OR for the highest versus lowest quartile was 1. 55 (95% CI: 1. 24 – 1. 95).

**Conclusion:**

There are positive results between LAP and depression after adjusting for multiple potential variables, and prospective studies are needed to verify the results.

## 1 Introduction

Depression is a type of mental illness characterized by low mood that interferes with an individual’s social functioning and leads to mental disability (1). Depression is a leading cause of disability, causing more than $10 billion a year in health care costs (2, 3). Depression is associated with diabetes (4), osteoarthritis (5), hypertension (6), obesity (7) and many other diseases, increasing the risk of hospitalization among depressed patients (8). An Australian study showed a biphasic relationship between depression and obesity, with a 37% increased risk of obesity among depressed people and an 18% increased risk of depression among obese people (9). This increased risk may be related to the presence of both genetic factors and lifestyle factors, such as a sedentary lifestyle, unhealthy diet, and smoking (10, 11). This dual correlation was stronger in the sensitivity analyses of young and middle-aged individuals.

Obesity is usually defined by relative body weight and is associated with a variety of cardiovascular and psychological disorders. Previously, BMI was often used to assess obesity, but in recent years, the accuracy of BMI in assessing obesity has been questioned (12). BMI does not reflect fat distribution or abdominal fat content and has limitations in assessing obesity (13). Excessive fat accumulation in patients with a normal BMI may also be hazardous to their health. Visceral fat accumulation increases the risk of depression (14) Yamamoto et al., 2016). Therefore, researchers have begun to study new indicators to assess obesity, and waist circumference(WC) is considered a better indicator because of its ability to assess abdominal fat accumulation (15) Fedewa et al., 2019). Researchers have introduced a new metric, the lipid accumulation product (LAP), which is an indicator of abdominal fat accumulation that is a combination of WC and triglyceride(TG) levels (16).

Several studies have shown that LAP is superior to WC and BMI in identifying CVD risk (16–18). In a longitudinal study on diabetes, LAP was shown to be associated with and better predict the 6-year incidence of type 2 diabetes than BMI (19). A German study showed that LAP was negatively associated with the risk of all-cause mortality (20). LAP is associated with a number of diseases, but few population-based epidemiological studies have examined the relationship between LAP and depression.

Moreover, whether subjects with higher LAP have an increased risk of depression is unknown. Therefore, the aim of our study was to examine the relationship between LAP and depression in adults in the general population.

## 2 Methods

### 2.1 Study design and participants

**Figure 1:**
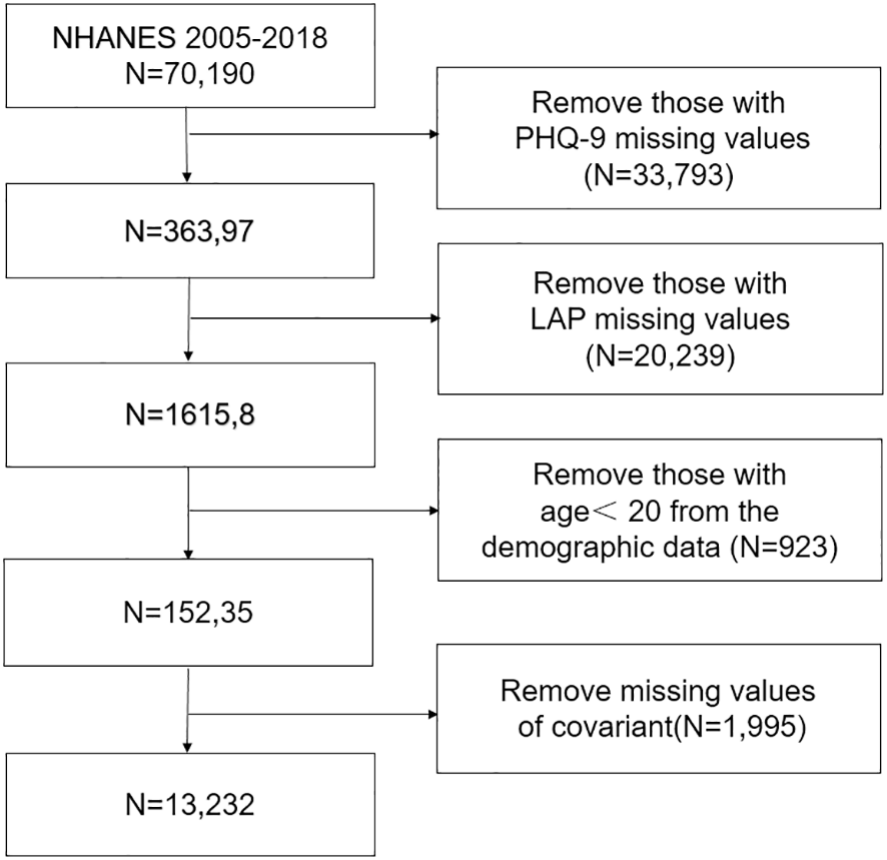
Selection of participants in the study.

The National Health and Nutrition Examination Survey (NHANES) is conducted by the National Center for Health Statistics (NCHS) of the Centers for Disease Control and Prevention to determine the status of nutrition and health in the United States. We analyzed data for eight cycles from 2005 to 2018. All participants signed informed consent forms, and the study protocol was approved by the NCHS. Specific ethics information is available on the website (NHANES - NCHS Research Ethics Review Board Approval (cdc.gov)). The exclusion criteria were as follows: age younger than 20 years or missing age, depression diagnosis, and LAP data. The detailed results are shown in the flow chart in **Figure 1**.

### 2.2 Variables

#### 2.2.1 Depression

In the NHANES dataset, the PHQ-9 was used to assess participants’ depressive symptoms over the past 2 weeks. Based on previous studies, a PHQ-9 score greater than 10 was defined as depression (21, 22).

#### 2.2.2 Assessment of LAP

Triglycerides in the NHANES are measured by fasting blood samples taken by health professionals at mobile screening centers and sent to professional institutions using the end point method in the Beckman Synchron LX system in mmol/L(NHANES 2005–2006: Standard Biochemistry Profile Data Documentation, Codebook, and Frequencies (cdc. gov). Waist circumference is measured by a health professional using a tape measure to measure the length above the iliac crest in the midaxillary line at the end of normal breathing, accurate to within 0. 1 cm (23). The formula for LAP is as follows:

LAP for men = (WC [cm] - 65) × (TG concentration [mmol/L])

LAP for women = (WC [cm] - 58) × (TG concentration [mmol/L]) (24).

#### 2.2.3 Covariates

The covariates included in our study were sex, race, age, marital status, education level, household income, body mass index, smoking status, alcohol consumption, diabetes status, and hypertension. Race was divided into four categories: Mexican American, non-Hispanic Black, non-Hispanic White and other. Education level was divided into four groups: college graduate or above, high school graduate/GED, below high school, or some college or AA degree. Marital status was divided into four groups: divorced/separated, married/cohabiting, never married, and widowed. BMI was classified as normal (18. 5≤BMI<25), overweight (25≤BMI<30) and obese (BMI≥30) (25). If there was a 0 in the group with a BMI <18. 5, the group was removed from the subgroup analysis. Age was divided into three groups: age group 1 (20–39), age group 2 (40–59) and age group 3 (≥60). Individuals were divided into three groups according to smoking status: never (smoked fewer than 100 cigarettes in a lifetime); former (current nonsmoker, former smoker who smoked more than 100 cigarettes); and current (smoking more than 100 cigarettes and regular smoking) smokers. Individuals were divided into three groups according to alcohol consumption: never (fewer than 12 drinks in a lifetime); former (drinking more than 12 drinks but not in the past year); and current (drinking more than 12 drinks in the past year) drinkers. The diagnostic criteria for diabetes were as follows: was diagnosed with diabetes by a doctor, was taking hypoglycemic medication and insulin, had a glycated hemoglobin level ≥6. 5%, had a fasting blood glucose level ≥7. 0 mmol/l, and had a random blood glucose or 2-hour OGTT blood glucose level ≥11. 1 mmol/l. The diagnostic criteria for hypertension were as follows: mean SBP≥140 mmHg or mean DBP≥90 mmHg; was diagnosed with hypertension by a doctor; and was taking blood pressure medication (26, 27).

### 2.4 Statistical analysis

Our analysis included NHANES sampling weights to increase the representativeness of the results. Continuous variables are expressed as the means ± standard errors, and categorical variables are expressed as percentages. LAP data were divided into quartiles, with the lowest quartile serving as the reference category. We used multiple weighted logistic regression models to analyze the association between LAP and depression, and the results are presented with 95% confidence intervals (CIs) and odds ratios (ORs). No variables were adjusted for in the crude model. Model 1 was adjusted for age, sex, and race. Model 2 was adjusted for marital status, household income, smoking status, alcohol consumption, hypertension status, and diabetes status based on Model 1. Model 3 further adjusted for BMI based on Model 2. In addition, subgroup analyses were conducted to test the association between LAP and depression by sex, race, diabetes status, and hypertension status. Finally, we conducted a sensitivity analysis and performed analysis of variance on the unweighted data. We included all covariates to perform inverse probability of treatment weighting (IPTW) on the unweighted data. R 4. 3. 0 was used for the statistical analysis. Restricted cubic splines (RCSs) were used to examine the nonlinear relationship between LAP and depression. All tests were two-sided, and p < 0. 05 was considered to indicate statistical significance.

## 3 Results

### 3.1 Patient characteristics

The NHANES database from 2005 to 2018 included 39,749 adult participants; 26,059 participants with missing values were excluded, and 13,240 individuals were included in the analysis. According to the unweighted data, 1098 (8. 29%) participants had depression, and 12,142 (91. 71%) did not have depression; similarly, the weighted ratios of depression to non-depression were 7. 16% and 92. 84%, respectively. As shown in **Table 1**, there were significant differences according to sex, race, household income, BMI, education level, marital status, smoking status, alcohol consumption, hypertension status, diabetes status, take statins, LAP and depression.

**Table 1:** Weighted baseline characteristics of patients with or without depression.

Variable	Total	Without depression	Depression	*P* value
Age(years)	47. 54 ± 0. 28	47. 53 ± 0. 30	47. 57 ± 0. 59	0. 95
Poverty	3. 05 ± 0. 04	3. 12 ± 0. 04	2. 12 ± 0. 08	< 0. 0001
BMI (kg/m^2^)	28. 98 ± 0. 10	28. 87 ± 0. 10	30. 46 ± 0. 26	< 0. 0001
WC	99. 33 ± 0. 25	99. 10 ± 0. 25	102. 21 ± 0. 66	< 0. 0001
TG	1. 43 ± 0. 02	1. 42 ± 0. 02	1. 60 ± 0. 05	< 0. 001
LAP	58. 13 ± 0. 90	57. 15 ± 0. 89	70. 82 ± 2. 60	< 0. 0001
Sex, %				< 0. 0001
Female	50. 08	49. 12	62. 51	
Male	49. 92	50. 88	37. 49	
Race, %				0. 004
Mexican American	8. 04	8. 08	7. 53	
Non-Hispanic Black	9. 86	9. 61	13. 14	
Non-Hispanic White	70. 21	70. 54	65. 89	
Other Race	11. 89	11. 77	13. 44	
Education, %				< 0. 0001
College graduate or above	30. 56	31. 9	13. 18	
High school graduate/GED or equivalent	23. 05	22. 75	27. 04	
less than high school	15. 14	14. 35	25. 39	
Some college or AA degree	31. 24	31	34. 38	
Marital, %				<0. 0001
Divorced/Separated	12. 81	11. 88	24. 79	
Married/Living with partner	64. 67	65. 9	48. 66	
Never married	17. 2	17. 01	19. 64	
Widowed	5. 33	5. 21	6. 91	
Hypertension, %				< 0. 0001
yes	37. 97	37. 15	48. 57	
no	62. 03	62. 85	51. 43	
DM, %				< 0. 0001
no	84. 21	84. 71	77. 7	
yes	15. 79	15. 29	22. 3	
Smoke, %				< 0. 0001
never	54. 06	55. 43	36. 34	
former	25. 74	25. 94	23. 02	
now	20. 2	18. 63	40. 64	
Drink, %				< 0. 001
never	10. 19	10. 22	9. 75	
former	13. 26	12. 88	18. 27	
now	76. 55	76. 9	71. 97	
Take statins, %				< 0. 0001
no	41. 04	42. 24	25. 47	
other	41. 32	40. 34	53. 96	
yes	17. 64	17. 42	20. 57	

### 3.2 Association of LAP with depression

The relationship between LAP and depression is shown in **Table 2**. The LAP was divided into four groups: Q1 (LAP =0. 34, 23. 75), Q2 (LAP =23. 75, 42. 60), Q3 (LAP =42. 60, 73. 60), and Q4 (LAP =73. 60, 2642. 84). No covariates were adjusted for in the crude model, and there was a positive relationship between LAP and depression. The OR with 95% CI in the highest quartile versus the lowest quartile was 2. 02 (1. 56, 2. 61). In Model 1, after adjusting for LAP, age, race and sex, the OR with 95% CI of depression in the highest quartile versus the lowest quartile was 2. 35 (1. 89, 2. 92). After further adjustment for marital status, PIR, educational level, smoking status, alcohol consumption, and hypertension and diabetes status based on Model 1, the OR with 95% CI in Model 2 was 1. 68 (1. 32, 2. 14). The relationship between LAP and depression was still robust in Model 3 (fully adjusted model; OR, 1. 50; 95% CI, 1. 05–2. 15). A significant positive trend was observed between increasing LAP and the risk of depression (p for trend<0. 05).

**Table 2:** Association between LAP and depression.

	crude Model	Model 1	Model 2	Model 3
LAP	OR (95% CI)	OR (95% CI)	OR (95% CI)	OR (95% CI)
Q1	ref	ref	ref	ref
Q2	1. 12(0. 80,1. 57)	1. 21(0. 92,1. 58)	1. 10(0. 83,1. 46)	1. 04(0. 77,1. 41)
Q3	1. 41(1. 04,1. 92)	1. 53(1. 17,2. 01)	1. 25(0. 94,1. 67)	1. 14(0. 81,1. 60)
Q4	2. 02(1. 56,2. 61)	2. 35(1. 89,2. 92)	1. 68(1. 32,2. 14)	1. 44(1. 01,2. 06)
p for trend	<0. 0001	<0. 0001	<0. 001	0. 02

Model 1: adjusted for age, sex and race;

Model 2: adjusted for age, sex, race, marital status, poverty, educational level, smoking status, alcohol consumption, hypertension, diabetes;

Model 3: further adjusted for BMI and take statins based on Model 2

### 3.3 Subgroup analyses

The results of the subgroup analyses are shown in **Table 3**. LAP was significantly positively associated with depression among males (2. 52, OR; 95% CI, 1. 39, 4. 57), non-Hispanic Black individuals (2. 55, OR; 95% CI, 1. 49, 4. 36), individuals without diabetes (1. 67, OR; 95% CI, 1. 06, 2. 61) and individuals in the overweight (2. 09, OR; 95% CI, 1. 23, 3. 54) subgroup.

**Table 3:** Subgroup analyses stratified by sex, race, BMI, age, diabetes and hypertension.

LAP	Q1	Q2	*p*	Q3	*p*	Q4	*p*	*p* for	*p* for
								trend	interaction
sex									0. 37
Female	ref	1. 03(0. 71,1. 49)	0. 9	1. 24(0. 75,2. 07)	0. 4	1. 32(0. 79,2. 21)	0. 28	0. 24	
Male	ref	1. 41(0. 81,2. 45)	0. 22	1. 38(0. 79,2. 42)	0. 25	2. 47(1. 34,4. 54)	0. 004	0. 001	
eth									0. 61
Non-Hispanic Black	ref	1. 49(0. 92,2. 41)	0. 1	1. 80(1. 04,3. 11)	0. 04	2. 47(1. 45,4. 22)	0. 001	0. 002	
Non-Hispanic White	ref	1. 23(0. 77,1. 96)	0. 39	1. 34(0. 75,2. 40)	0. 31	1. 57(0. 89,2. 78)	0. 12	0. 12	
Other Race	ref	0. 91(0. 54,1. 54)	0. 73	1. 14(0. 63,2. 08)	0. 66	1. 48(0. 77,2. 85)	0. 23	0. 13	
Mexican American	ref	0. 80(0. 34, 1. 90)	0. 61	0. 68(0. 31, 1. 50)	0. 33	1. 60(0. 69, 3. 72)	0. 27	0. 01	
BMI									0. 49
obese	ref	0. 93(0. 48,1. 81)	0. 84	1. 01(0. 49,2. 06)	0. 99	1. 11(0. 56,2. 21)	0. 76	0. 38	
normal	ref	1. 33(0. 82,2. 17)	0. 24	1. 58(0. 72,3. 44)	0. 25	1. 91(0. 73,4. 98)	0. 19	0. 1	
overweight	ref	1. 00(0. 59,1. 69)	1	1. 10(0. 66,1. 84)	0. 7	2. 08(1. 22,3. 56)	0. 01	0. 001	
age									0. 86
age1	ref	0. 91(0. 55,1. 51)	0. 71	1. 22(0. 70,2. 12)	0. 49	1. 47(0. 85,2. 56)	0. 17	0. 06	
age2	ref	1. 31(0. 69,2. 46)	0. 4	1. 06(0. 57,1. 97)	0. 84	1. 56(0. 76,3. 21)	0. 22	0. 21	
age3	ref	1. 33(0. 76, 2. 34)	0. 31	1. 38(0. 61, 3. 12)	0. 44	1. 60(0. 75, 3. 39)	0. 22	0. 24	
Hypertension									0. 39
yes	ref	0. 89(0. 52,1. 54)	0. 68	1. 20(0. 73,1. 99)	0. 47	1. 64(0. 93,2. 88)	0. 09	0. 004	
no ref		1. 32(0. 87,2. 00)	0. 2	1. 31(0. 77,2. 21)	0. 32	1. 44(0. 88,2. 38)	0.15	0.11	
Diabetes									0.07
yes	ref	1. 22(0. 84,1. 78)	0. 28	1. 44(0. 91,2. 27)	0. 12	1. 59(1. 01,2. 51)	0.28	0.01	
no	ref	0. 87(0. 38,2. 00)	0. 74	0. 86(0. 37,1. 97)	0. 71	1. 48(0. 71,3. 09)	0.03	0.05	

Adjusted for age, sex, race, marital status, poverty, educational level, smoking status, alcohol consumption, hypertension, diabetes, take statins and BMI except the stratification factor itself.

### 3.4 Sensitivity analyses

The results of the subgroup analysis are presented in **Table 4**. After IPTW, the OR for the highest quartile versus the lowest quartile was 1. 55 (95% CI: 1. 24–1. 95). The trend test yielded results consistent with those of the previous analysis (p for trend<0. 01).

**Table 4:** Sensitivity analyses.

LAP	OR (95CI)	*p* for trend
Inverse probability treatment weighted analyses		
Q1	ref	
Q2	0. 99(0. 81,1. 22)	
Q3	1. 10(0. 89,1. 37)	
Q4	1. 55(1. 24,1. 95)	<0. 01

adjusted for age, sex, race, marital status, poverty, educational level, smoking status, alcohol consumption, hypertension, diabetes, and BMI.

### 3.5 The non-linear relationship between LAP and depression

**Figure 2:**
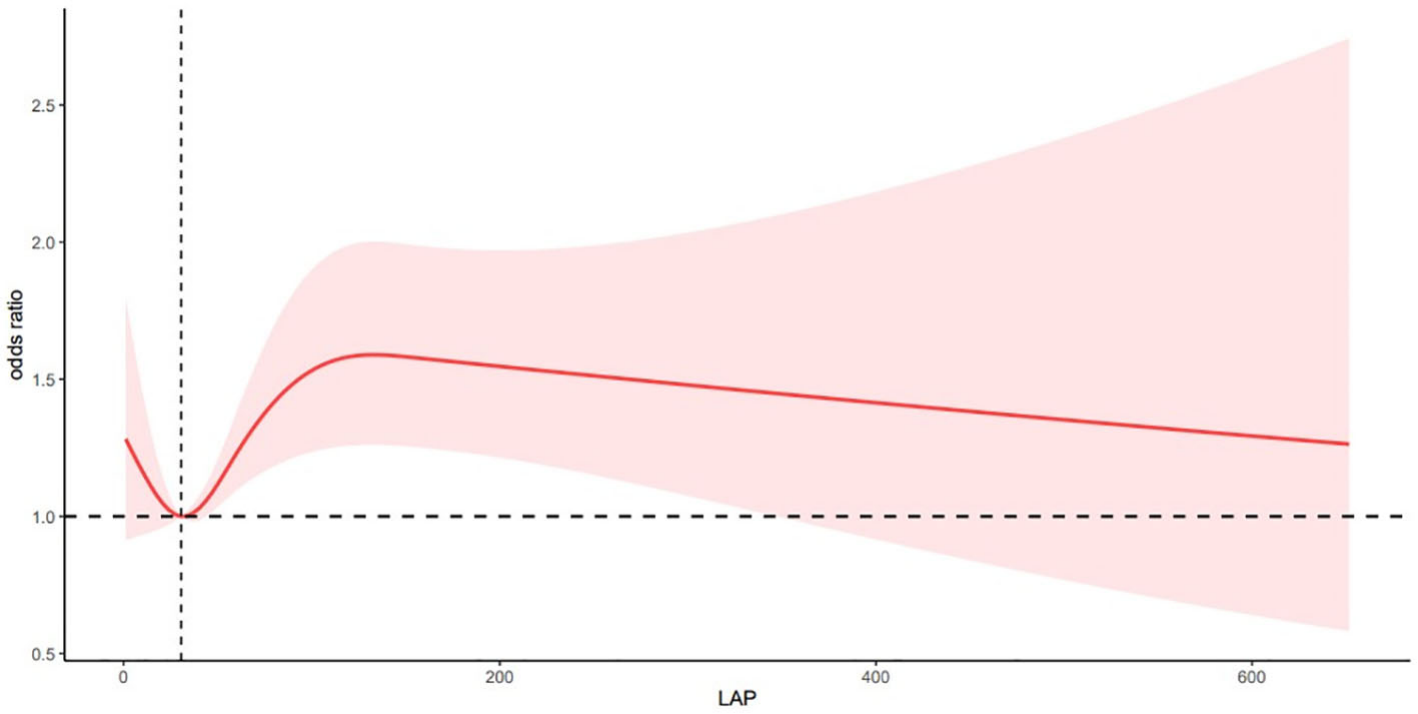
Association of LAP and depression in a RCS model among all participants.

After adjusting for multiple variables, the RCS analysis showed a nonlinear correlation between LAP and depression (P for nonlinearity < 0. 05; **Figure 2**). The risk of depression was significantly lower when the LAP value was <30. 56, significantly higher when the LAP value was in the range of 30. 56–131. 90, and slightly lower when the LAP value was greater than 131. 90. There was a complex nonlinear relationship between Lap and depression.

*P* overall< 0. 05 P for nonlinear< 0. 05

Adjusted for age, sex, race, marital status, poverty, educational level, smoking status, alcohol consumption, hypertension, diabetes, and BMI.

## 4 Discussion

To our knowledge, this is the first study to explore the relationship between LAP and depression using a representative sample from the United States. We found a positive association between LAP and depression. Subgroup analyses revealed higher ORs for the association between LAP and depression in men, non-Hispanic Black people, individuals without diabetes, and overweight individuals. The results of the sensitivity analysis supported our findings.

To date, there are limited data on the relationship between LAP and depression. The LAP was originally proposed by Kahn et al. to compensate for the inadequacy of BMI in assessing obesity (24). In a cross-sectional sample of 8942 middle-aged and elderly people in China, researchers found that LAP was correlated with depressive symptoms in men and women and could be used as an indicator of depressive symptoms in middle-aged and elderly people (28). The results for middle-aged and older Asians may not be representative of the American population. In addition, adjusting for BMI made our results on LAP more reliable.

The mechanism of the relationship between LAP and depression needs to be further explored and may be related to the following reasons. First, depression is associated with insulin resistance (IR) (29, 30). IR levels increase by 6. 1% in women and 13. 2% in men with depression, and WC partially mediates this association (31). Animal studies have shown that IR alters dopamine transport in mice, leading to depression-like behavior (32). Curcumin can upregulate insulin receptor substrates in the liver, improve insulin sensitivity, and alleviate depression-like behavior in rats (33). In the population, high glycemic variability increases the incidence of depression (34). LAP is a novel indicator of IR (35). Our study is the first to observe a positive association between LAP quartiles and depression in a representative population. A high LAP may also mediate depression in a manner similar to IR. Second, triglycerides may bind to glucocorticoid receptors via cortisol, and glucocorticoid receptors activate lipids to inhibit lipid mobilization, thereby affecting metabolic abnormalities of the hypothalamic pituitary adrenal axis and contributing to depression (36, 37). Finally, a greater WC causes abdominal fat to accumulate as white fat in the form of stored triglycerides, and adipose tissue secretes cytokines such as IL-6 and TNF-α (38, 39). Animal studies have shown that IL-6 and TNF-α lead to depressive behavior in mice through neuroinflammatory pathways (40–42).

According to our subgroup analysis, a higher LAP was associated with a greater risk of depression in men (p for trend < 0. 05). This finding may be related to higher rates of IR among men than among women (31), and longitudinal studies have shown that IR increases the risk of developing depression 5 years later (43). This difference may also be related to differences in leptin levels between men and women among depressed patients (44, 45). In addition, WC is higher in men than in women (46). Women store most of their fat in subcutaneous fat such as hips and thighs, while male fat is concentrated in the viscera and is associated with high central obesity (47). Estrogen is lower in men than in women and estrogen is protective against fat accumulation (48) In the overweight population, a higher LAP was associated with a greater risk of depression. Studies have shown that overweight, depressed patients may have increased exogenous peripheral inflammation, and decreased systemic inflammation contributes to the development of depression (49, 50). Therefore, it is important to consider men with a high LAP and overweight as a high-risk group for depression. Further clinical and longitudinal studies are needed to validate our findings.

The strengths of our study include the fact that the results can be extrapolated to the general population based on a large sample population and a sophisticated sampling design. Second, the collection of laboratory, scale assessment and anthropometric data was standardized and homogeneous. Third, the LAP test is relatively convenient and inexpensive, making it easier to generalize its use to clinical settings in the future. However, this study also has several limitations: this was a cross-sectional study, and a causal relationship between LAP and depression could not be inferred. Second, the diagnosis of depression was made using the PHQ-9 scale rather than the gold standard of the Diagnostic and Statistical Manual of Mental Disorders (DSM-V), but numerous studies have demonstrated the reliability of the PHQ-9 (22, 51). Finally, the influence of antilipidemic drugs could not be excluded.

## 5 Conclusion

In conclusion, our study revealed a significant association between LAP and depression after we adjusted for multiple confounders. Lowering LAP may play an important role in the diagnosis and treatment of depression, but this needs to be further confirmed in prospective studies.

## Data availability statement

Publicly available datasets were analyzed in this study. This data can be found here: www.cdc.gov/nchs/nhanes/index.htm.

## Ethics statement

The studies involving humans were approved by NCHS Research Ethics Review Board Approval. The studies were conducted in accordance with the local legislation and institutional requirements. The participants provided their written informed consent to participate in this study.

## Author contributions

XZ: Conceptualization, Data curation, Software, Writing – original draft, Writing – review & editing. PW: Conceptualization, Data curation, Software, Writing – original draft, Writing – review & editing. YY: Writing – original draft, Writing – review & editing. TW: Writing – original draft, Writing – review & editing. JC: Writing – original draft, Writing – review & editing. YS: Conceptualization, Funding acquisition, Supervision, Writing – review & editing. LM: Conceptualization, Supervision, Writing – review & editing.
